# Efficacy and Safety of Probiotics Combined With Traditional Chinese Medicine for Ulcerative Colitis: A Systematic Review and Meta-Analysis

**DOI:** 10.3389/fphar.2022.844961

**Published:** 2022-03-07

**Authors:** Yu Hu, Zhen Ye, Yingqi She, Linzhen Li, Mingquan Wu, Kaihua Qin, Yuzheng Li, Haiqing He, Zhipeng Hu, Maoyi Yang, Fating Lu, Qiaobo Ye

**Affiliations:** ^1^ School of Basic Medical Sciences, Chengdu University of Traditional Chinese Medicine, Chengdu, China; ^2^ Hospital of Chengdu University of Traditional Chinese Medicine, Chengdu, China; ^3^ Department of Pharmacy, Sichuan Provincial Orthopedic Hospital, Chengdu, China; ^4^ Health Preservation and Rehabilitation College, Chengdu University of Traditional Chinese Medicine, Chengdu, China

**Keywords:** treatment, traditional Chinese medicine, meta-analysis, systematic review, ulcerative colitis (colitis ulcerosa), probiotic

## Abstract

**Background:** The combination of probiotics and traditional Chinese medicine (TCM) is a prospective therapy for ulcerative colitis (UC), and its efficacy and safety need to be urgently evaluated.

**Objective:** This study aims to comprehensively assess the efficacy and safety of probiotics combined with TCM for the treatment of UC.

**Methods:** The Pubmed, EMBASE, Cochrane library, China Academic Journals (CNKI), Wan-fang database, Chinese biomedical literature service system (CBM), and Chinese Science and Technology Journals (CQVIP) were searched. Subgroup analysis were designed in accordance with different control drugs, treatment courses, and types of probiotics. The Review Manager software (version 5.4.1) was utilized for statistical analysis.

**Results:** 14 original studies containing 1,154 patients were analyzed and showed that probiotics with TCM was more effective than 5-aminosalicylic acid (5-ASA), probiotics or TCM used individually. Moreover, probiotics combined with TCM could inhibit the intestinal inflammation, reduce the recurrence rate and the incidence of adverse events. The subgroup analysis showed that a mixture of different probiotics was more effective than a single strain.

**Conclusion:** It is suggested that probiotics combined with TCM could effectively control clinical symptoms, inhibit intestinal inflammatory response, and finally slow down the disease progress and reduce the disease recurrence with less adverse events. The mixture of different probiotics used in conjunction with individually tailored TCM is a potential clinical strategy for UC.

## Introduction

Ulcerative colitis (UC) is a chronic inflammatory bowel disease (IBD) that caused by inappropriate immune responses to intestinal commensal microbes (Abraham and Kane, 2012; Ng et al., 2013). It is characterized by chronic recurring inflammation, damage to the quality of life of patients and is a risk factor for colorectal cancer (Kirsner, 1979; Ordás et al., 2012; Ungaro et al., 2017). UC had been dubbed a “Western disease” due to its significant prevalence in industrialized countries and regions such as Europe, Oceania, and the United States. At present, the annual incidence of UC is approximately 8.8–23.1 cases per 100,000 people in North America, 0.6–24.3 cases per 100,000 people in Europe, and 7.3–17.4 cases per 100,000 people in Oceania (Du and Ha, 2020). Although the incidence of UC in developed countries and regions has begun to stabilize since the 21st century, the incidence of UC has begun to rise significantly in regions with more emerging industrialized countries such as in Asia and South America due to the changes in living and dietary structure (Kaplan and Ng, 2016; Mak et al., 2020). As the most populous country, the annual incidence rate of UC in China is about 1.2 cases per 100,000 people and shows a steady upward trend (Wei et al., 2021). Consequently, the global incidence of UC continues to increase rapidly, making it a global disease (Kaplan, 2015; Kaplan and Ng, 2017).

Immunological abnormalities are considered to play an important role in UC (Nascimento et al., 2020), and drugs regulating innate immune system have become mainstream treatment. 5-aminosalicylic acid (5-ASA), corticosteroids, and thiopurines can effectively alleviate UC as first-line therapeutic agents. Unfortunately, intolerance or non-response to drugs, adverse events, and toxicity prevent the UC patients from getting remission. Notably, the gut microbiota also plays an important role in the host immune response. Therefore, therapeutic approaches aimed at modifying gut microbial dysbiosis are being explored and assessed as prospective therapy strategies for UC (Celiberto et al., 2015; Li Q. et al., 2020). Probiotics are active microorganisms which can have a positive health impact on the body when consuming in sufficient doses (Astó et al., 2019). By regulating the microbial balance, probiotics are believed to be able to repair the disrupted microbiota in UC patients (Kamarli Altun et al., 2019). In order to evaluate the potential of probiotics in the treatment of UC, various preclinical and clinical trials have been conducted. Some probiotic strains, such as *Akkermansia muciniphila*, *Roseburia intestinalis* and *Bifidobacterium infantis*, can improve acute and chronic experimental colitis by regulating the microbiota and inhibiting the inflammatory response (Bian et al., 2019; Han et al., 2021; Xu et al., 2021). Similarly, the probiotic products Symprove and VSL#3, also can regulate microbial dysbiosis and restore barrier function in UC patients (Ghyselinck et al., 2020). Some experts believe that probiotics should be considered not only a simple dietary supplement, but also a potential therapeutic factor for UC (Filidou and Kolios, 2021).

However, the application of probiotics individually does not always achieve excellent clinical outcomes (Yoshimatsu et al., 2015). *Bifidobacterium breve*, the same probiotic strain was used in two clinical studies for the treatment of UC patients, but one of them was terminated due to a lack of therapeutic effect, suggesting the instability of probiotic efficacy (Groeger et al., 2013; Matsuoka et al., 2018). In addition, multiple systematic evaluations have concluded that the evidence does not support the effectiveness of probiotics in maintaining remission of UC, which means that it is difficult to use probiotics alone as a sufficient treatment option for UC (Naidoo et al., 2011; Iheozor-Ejiofor et al., 2020). Interestingly, studies have shown that traditional Chinese medicine (TCM) has the therapeutic value for UC by inhibiting abnormal inflammatory response, protecting intestinal barrier, and restoring gut microbiota imbalance (Zhang et al., 2013). When integrated into therapeutic plan, TCM is believed to improve the efficacy and reduce the side effects of drugs (Li Y. et al., 2020). Furthermore, probiotics combined with TCM are widely applied in UC in China. Therefore, it is of great significance to further evaluate the clinical value of probiotics combined with TCM based on evidence-based medicine. In this study, the efficacy and safety of probiotics combined with TCM were evaluated through systematic evaluation and meta-analysis, and the potential mechanism was summarized to provide reference for clinical rational drug use.

## Materials and Methods

This study followed the Preferred Reporting Items for Systematic Reviews and Meta-analysis (PRISMA) statements checklist (Page et al., 2021).

### Study Selection

Pubmed, EMBASE, Cochrane library, China Academic Journals (CNKI), Wan-fang database, Chinese biomedical literature service system (CBM), Chinese Science and Technology Journals (CQVIP), and other databases were systematically searched from the founding to November, 2021. No language constraints were considered for publications. The search keywords were following: “traditional Chinese medicine OR Chinese medicine OR Herbal medicine OR Herb” AND “Probiotic OR Probiotics” AND “Ulcerative colitis.”

### Inclusion and Exclusion Criteria

Studies were included if they met the PICOS criteria. Specifically, patients with a definite diagnosis of UC were included in the study and those with Crohn’s disease or unspecified IBD subtypes were excluded. The original literature was included only if probiotics combined with TCM were used a mean of intervention, and excluded if other therapeutic measures were added. To comprehensively assess whether the combined use of probiotics and TCM is effective and necessary, original studies were included, including 5-ASA or probiotics or TCM alone as control measures. The clinical effective rate is the primary outcome in this meta-analysis. The recurrence rate, inflammatory cytokines of proteins, including C-reaction protein (CRP), tumor necrosis factor-α (TNF-α), Interleukin (IL)-6, IL-8, IL-10 and adverse events are the secondary outcomes. Primary outcome must be reported in the original literature, while secondary outcomes are not mandatory. At last, only the randomized controlled trials (RCTs) were included for analysis.

### Data extraction

The included studies were reviewed independently by two authors (ZY and YS) to extract the following data: first author, publication year, number of patients, probiotic species, control drugs, course, outcomes, follow-up times and adverse events. Any differences were resolved through discussion between the two authors.

### Risk of Bias Assessment

The Cochrane risk of bias tool was performed to assess the risk of bias of the included studies (Higgins et al., 2011). The risk of bias was comprised of seven different domains: random sequence generation, allocation concealment, blinding of participants and personnel, blinding of outcome assessment, incomplete outcome data, selective outcome reporting and other sources of bias. The specific risks of bias are classified as high risk, low risk, and unclear risk of bias.

### Outcomes

The primary outcome was the clinical effective rate. In order to clarify the therapeutic potential of probiotics and TCM and provide theoretical support for their practical application, we set up subgroups analysis of clinical effective rate of different control drugs, treatment courses, and different types of probiotic species. The secondary outcomes were concluded recurrence rate, inflammatory cytokines (CRP, TNF-α, IL-6, IL-8, IL-10) and adverse events. Similarly, the subgroup analysis of recurrence rate in different courses was set to analyze the effectiveness of long-term use of probiotics combined with TCM. In addition, subgroups analysis of adverse events was set up to further evaluate the safety of probiotics combined with TCM with the course of treatment and specific symptoms as classification criteria.

### Statistical Analysis

The Review Manager software (version 5.4.1) was utilized for statistical analysis. The risk ratio (RR) and 95% confidence interval (CI) were used to analyze the dichotomous variables. The standardized mean difference (SMD) and a 95% CI were applied to assess the continuous variables. Heterogeneity was estimated by Cochran’s Q test and assessed using I^2^. When I^2^>50%, it is considered to have significant heterogeneity and a random-effects model is used to estimate the pooled effect. In contrast, a fixed-effects model was used. *p* < 0.05 was considered significant. The funnel plot was conducted to the evaluation of publication bias.

## Results

### Study Research

**FIGURE 1:**
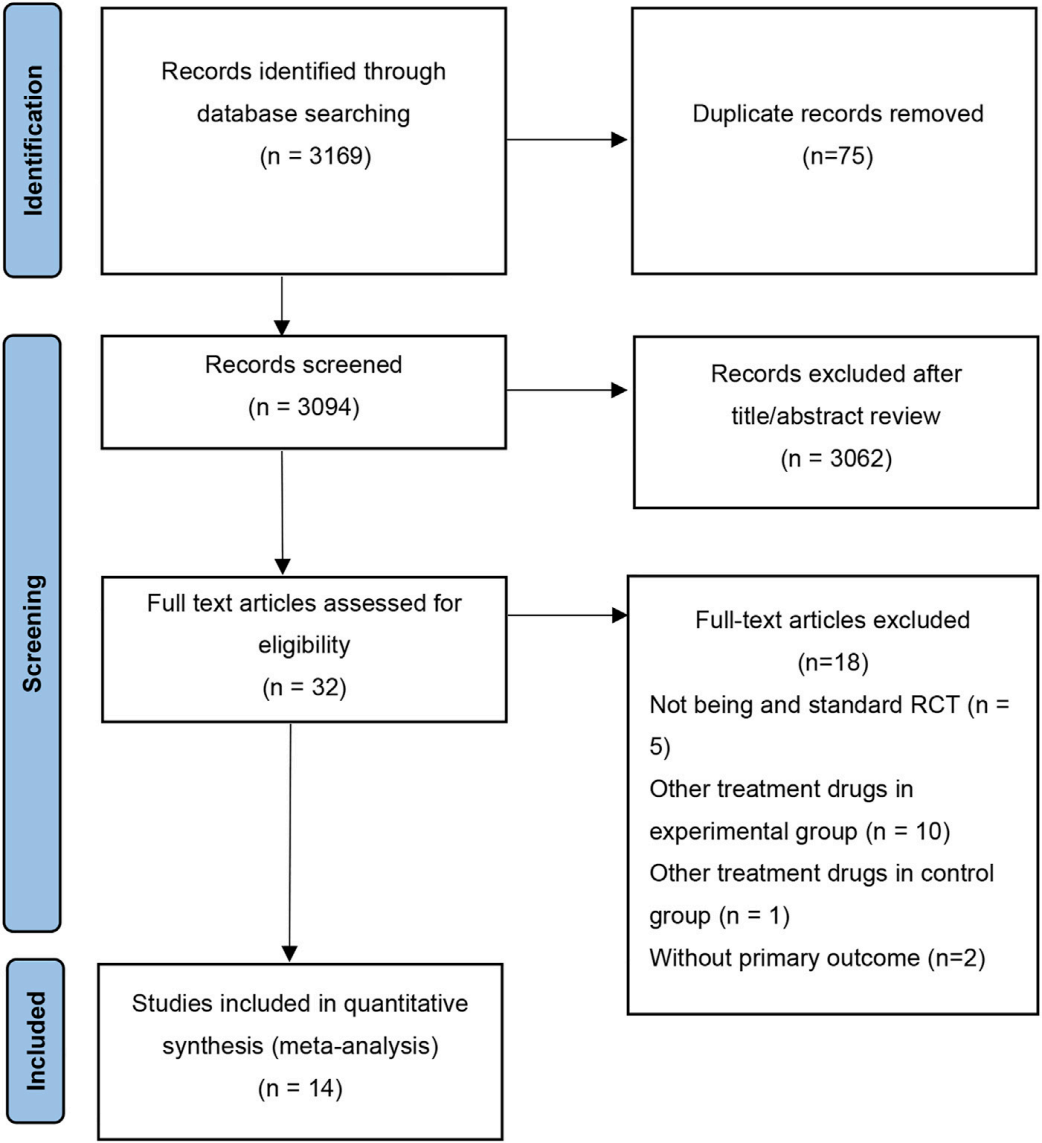
The flow of original literature selection.

As shown in Figure 1, a total of 3,169 studies were obtained by searching the databases. After excluding duplicate literature, 3,094 articles were remained. 32 articles were retained after reviewing the titles and abstracts. Finally, 18 studies that did not meet the criteria were excluded by reviewing the full text, and 14 studies were finally retained for this meta-analysis.

### Study Characteristics

The general characteristics of 14 included studies were shown in Table 1. The published raged from 2008 to 2019, a total of 1,154 patients were included, with 584 in the treatment group and 570 in the control group. 5-ASA was selected as the control group measure in six studies, probiotics were used alone in three studies, and TCM were used alone as control measures for five studies. All 14 studies were conducted in China.

**TABLE 1:** The characteristics of included study in this meta-analysis.

Study	The number of patients		Probiotics used	Control	Course	Outcomes	Follow-up	Adverse events
	Experimental group	Control group						
Gong 2019 (Gong, 2019)	24	16	(1) (2)	Probiotics	8 weeks	(a) (c) (g)	NR	NR
Ge et al., 2019 (Ge et al., 2019)	86	86	(2) (3) (4) (5)	TCM	8 weeks	(a) (b) (c) (d) (e) (f)	8 weeks	NR
Zhao et al., 2019 (Zhao et al., 2019)	63	63	(2) (3) (4)	TCM	8 weeks	(a) (c) (d)	NR	NR
Wang et al., 2018a (Wang and Liu, 2018)	48	48	(1) (2)	5-ASA	1 month	(a) (b) (h)	1 year	E:0 C:5
Li 2018 (Li, 2018)	46	46	(2) (3) (4)	Probiotics	4 weeks	(a) (c) (e) (f) (g)	NR	E:0 C:0
Chen et al., 2017 (Chen et al., 2017)	28	25	(1) (2)	5-ASA	1 month	(a) (c) (h)	1 year	E:0 C:4
Zhu 2015 (Zhu, 2015)	44	44	(2) (3) (4)	TCM	4 weeks	(a) (c) (e) (f) (g)	NR	E:0 C:0
Zhang 2015 (Zhang, 2015)	37	37	(6)	TCM	15 days	(a) (b)	NR	NR
Guo et al., 2014 (Guo et al., 2014)	40	40	(2) (3) (4) (5)	Probiotics	3 months	(a) (h)	NR	E:2 C:0
Xu 2013 (Xu, 2013)	50	50	(2) (3) (4) (5)	5-ASA	3 weeks	(a) (b)	3 months	NR
Chen et al., 2010 (Chen et al., 2010)	38	35	(4) (7) (8)	5-ASA	3 months	(a) (b) (h)	3 months	E:1 C:11
Zhang et al., 2010 (Zhang et al., 2010)	30	30	(6)	5-ASA	1 month	(a)	NR	NR
Gao et al., 2010 (Gao et al., 2010)	20	20	(6)	TCM	8 weeks	(a) (b)	6 months	E:0 C:0
Lu et al., 2008 (Lu et al., 2008)	30	30	(2) (3) (4)	5-ASA	4 weeks	(a) (h)	NR	E:1 C:9

(1) *Bacillus subtilis* (2) *Enterococcus Faecium* (3) *Bifidobacterium infantis* (4) *Lactobacillus acidophilus* (5) *Bacillus cereus* (6) *Bacillus licheniformis* (7) *Lactobacillus lactis* (8) *Lactococcus lactis subsp. Lactis*. (a) Clinical effective rate (b) Recurrence rate (c) TNF-α (d) IL-6 (e) IL-8 (f) IL-10 (g) CRP (h) adverse events. NR, not reported; TCM, traditional Chinese medicine; E, experimental group; C, control group.

### Risk of Bias Evaluation

**FIGURE 2:**
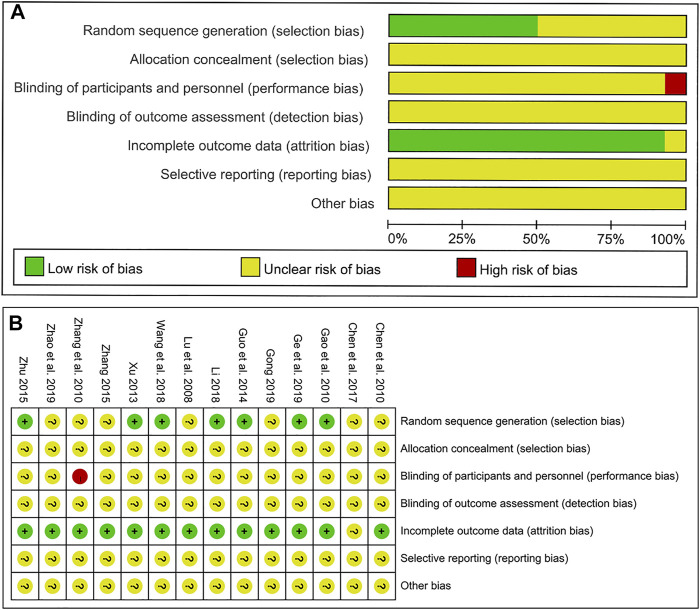
Results of the risk of bias assessment of the original literature included in the meta-analysis. **(A)** Overall risk of bias assessment results. **(B)** Risk of bias results for each specific original study.

As shown in Figure 2, we assessed the risk of bias for each study by the Cochrane risk of bias tool. For random sequence generation, seven studies mentioned “random” and described the specific random assignment method and were therefore judged as low risk. The remaining seven studies were classified as an unclear risk because they only mentioned randomization but did not specify the method used. For allocation concealment, all studies were assessed as unclear because of the absence of detailed information. For blinding of participants and personnel, 13 studies were regarded as unclear due to the lack of detailed description. However, one study referred to the treatment based on syndrome differentiation, which means that the selection of different herbal medicinals depending on the specific symptoms of patients, is considered to be of high risk. For blinding of outcome assessment, all studies were assessed as unclear risk due to the lack of details. For incomplete outcome data, one trial was judged to be unclear risk because the authors did not explain the specific reasons why patients were lost to follow-up. Finally, all studies regarding selective outcome reporting and other sources of bias were considered to be of unclear risk as no specific information as described.

### Meta-Analysis of Clinical Effective Rate

**FIGURE 3:**
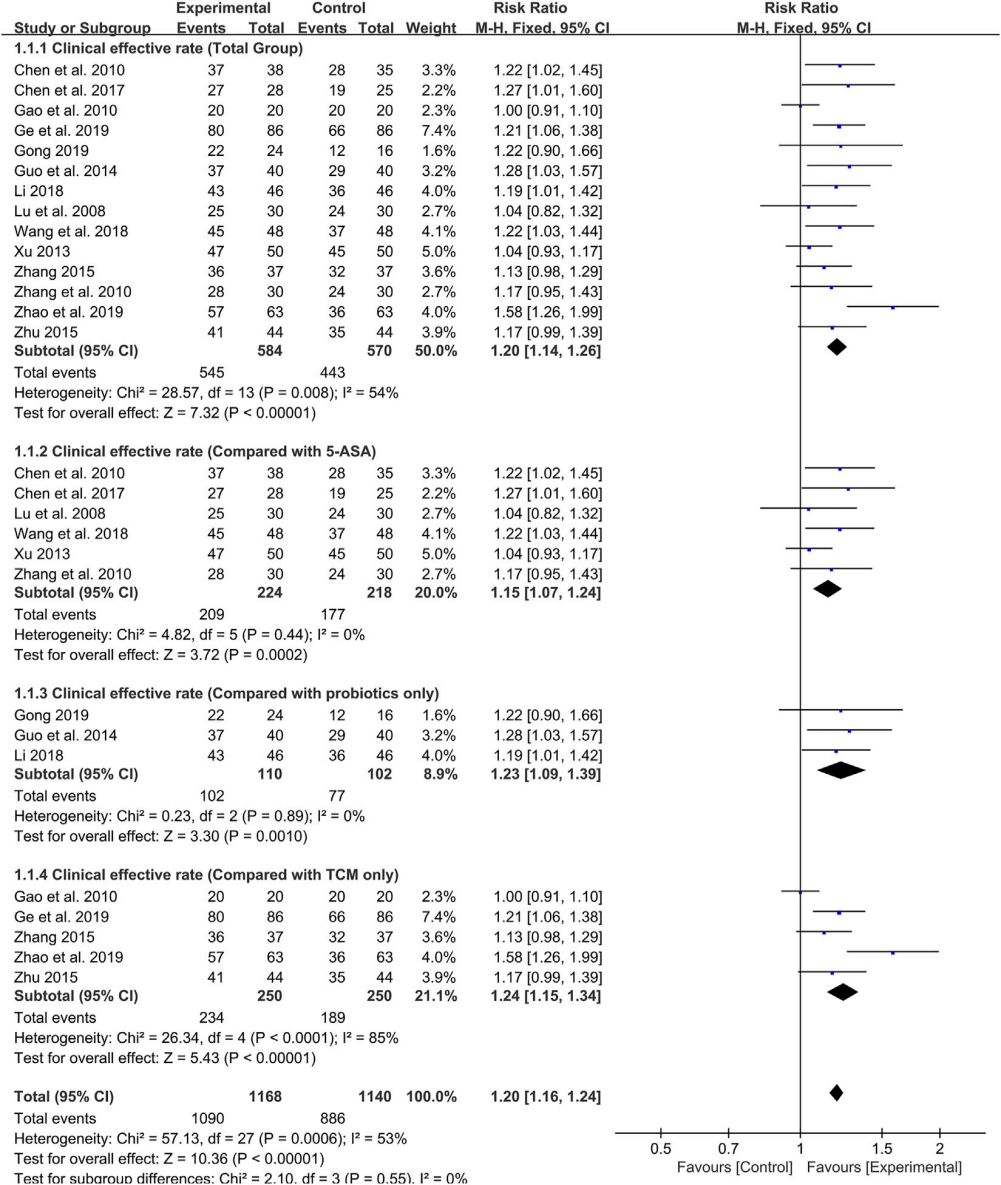
Forest plot displaying the results of the meta-analysis for clinical effective rate in probiotics combined with TCM group vs. different controlled drugs group.

As the primary outcome, the clinical effective rate was reported in all 14 included studies. Evaluated by a random-effects model, the meta-analysis results showed that the clinical efficiency of probiotics combined with TCM group was significantly higher than that of the control group (RR: 1.20; 95% CI: 1.14, 1.26; *p* < 0.00001, I^2^ = 54%) (Figure 3).

As different therapeutic drugs were selected for the control group in included studies, we set up subgroup analysis to further evaluate the efficacy of probiotics combined with TCM. 5-ASA was used in six studies, and the results showed that the clinical effective rate of probiotics combined with TCM group was higher than that of 5-ASA group (RR: 1.15; 95% CI: 1.07, 1.24; *p* = 0.0002, I^2^ = 0%) (Figure 3). Additionally, three studies reported that probiotics were used alone in the control group. The results also showed that the clinical effective rate of probiotics combined with TCM group was higher than that of probiotics group (RR: 1.23; 95% CI: 1.09, 1.39; *p* = 0.001, I^2^ = 0%) (Figure 3). In the remaining five studies, TCM was used alone as the treatment for the control group. Although there was high heterogeneity, the results still indicated that the probiotic combined with TCM group possessed a higher clinical effective rate compared with TCM group (RR: 1.24; 95% CI: 1.15, 1.34; *p* < 0.00001, I^2^ = 85%) (Figure 3).

**FIGURE 4:**
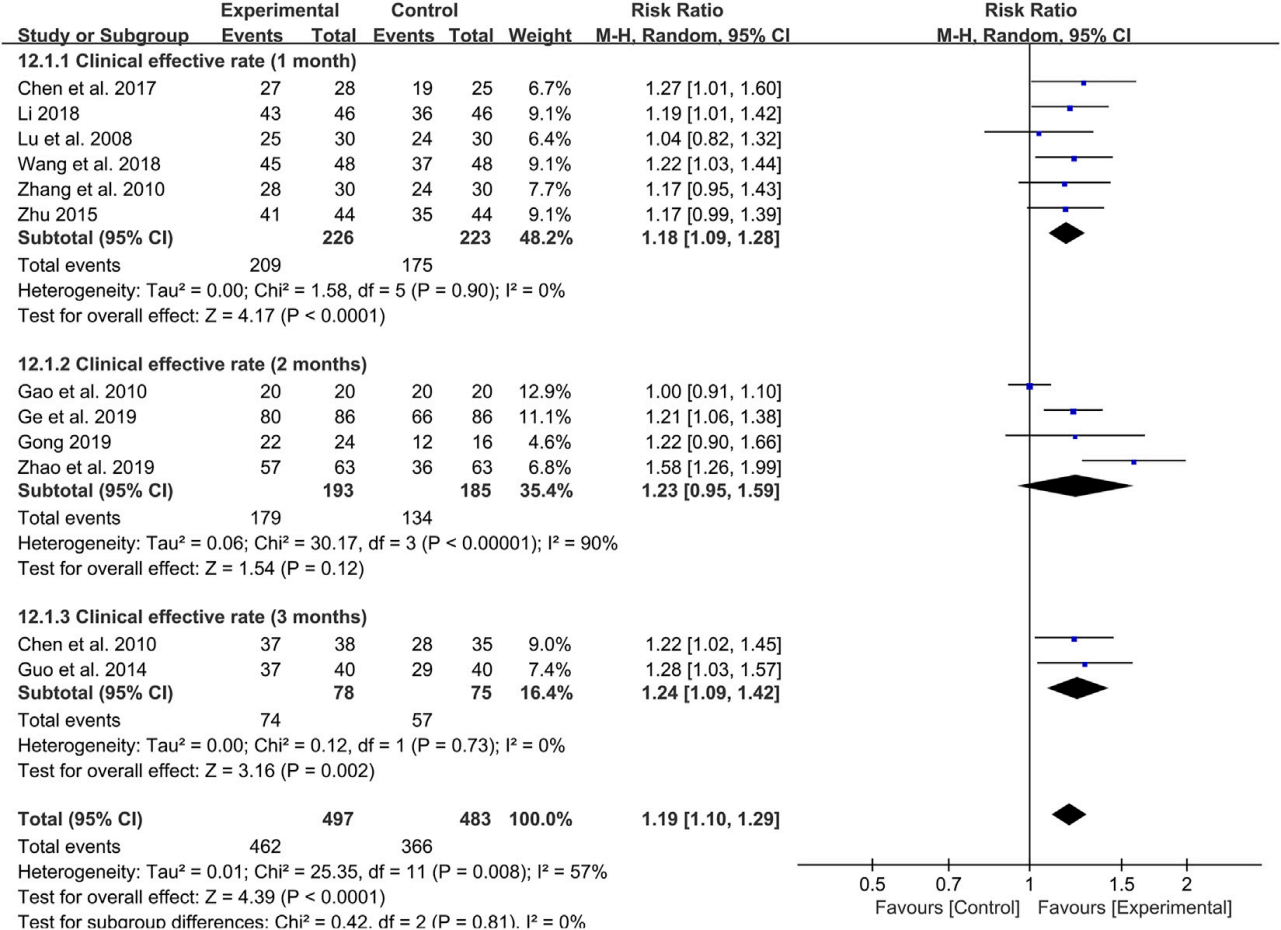
Forest plot displaying the results of the meta-analysis for clinical effective rate in different course of treatment.

Furthermore, we also set up subgroup analysis based on specific treatment courses. Before the analysis, the 4-week treatment course was categorized as 1 month and the 8-week course was viewed as 2 months for subgroup analysis. Six studies were treated with probiotics combined with TCM for 1 month. Meta-analysis showed that the effective rate was significantly higher than that of the control group (RR: 1.18; 95% CI: 1.09, 1.28; *p* < 0.0001, I^2^ = 0%) (Figure 4).

Likewise, the 3-month treatment course also showed a high clinical effective rate of probiotics combined with TCM in two studies (RR: 1.24; 95% CI: 1.09, 1.42; *p* = 0.002, I^2^ = 0%) (Figure 4). It is worth noting that there is no difference in the clinical effective rate between probiotics combined with TCM and control group in four studies with 2 months as treatment course (RR: 1.23; 95% CI: 0.95, 1.59; *p* = 0.12, I^2^ = 90%) (Figure 4).

**FIGURE 5:**
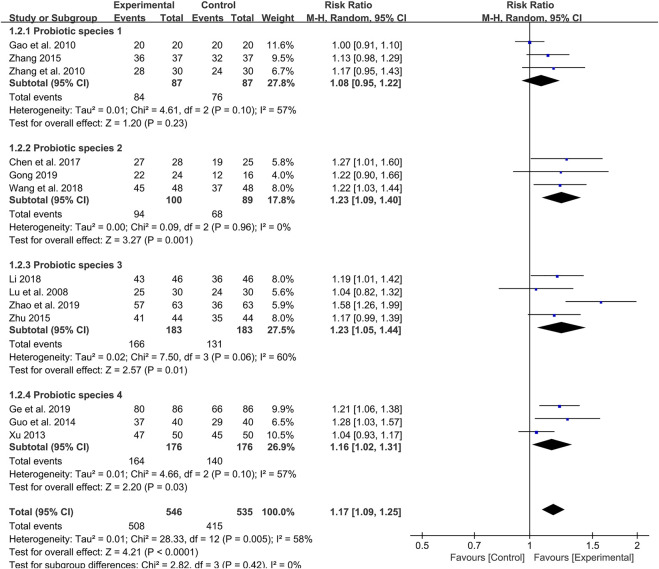
Forest plot displaying the results of the meta-analysis for clinical effective rate in different probiotics species used.

Among the 14 included studies, the species of probiotic used were also different (Table 1). Therefore, a subgroup analysis was also conducted to evaluate whether the clinical effective rate was affected by the different species of probiotics used. The probiotic species 1 (*Bacillus licheniformis)* was used in three studies, but there was no difference in clinical effective rate between the probiotics combined with TCM group and control group (RR: 1.08; 95% CI: 0.95, 1.22; *p* = 0.23, I^2^ = 57%) (Figure 5). However, the results indicated that the probiotics combined with TCM enhanced the clinical efficiency with probiotics species 2 (*Bacillus subtilis* and *Enterococcus Faecium*) (RR: 1.23; 95% CI: 1.09, 1.40; *p* = 0.001, I^2^ = 0%) (Figure 5), species 3 (*Enterococcus Faecium*, *Bifidobacterium infantis* and *Lactobacillus acidophilus*) (RR: 1.23; 95% CI: 1.05, 1.44; *p* = 0.01, I^2^ = 60%) (Figure 5) and probiotics species 4 (*Enterococcus Faecium*, *Bifidobacterium infantis*, *Lactobacillus acidophilus* and *Bacillus cereus*) (RR: 1.16; 95% CI: 1.02, 1.31; *p* = 0.03, I^2^ = 57%) (Figure 5) used.

### Meta-Analysis of Recurrence Rate

**FIGURE 6:**
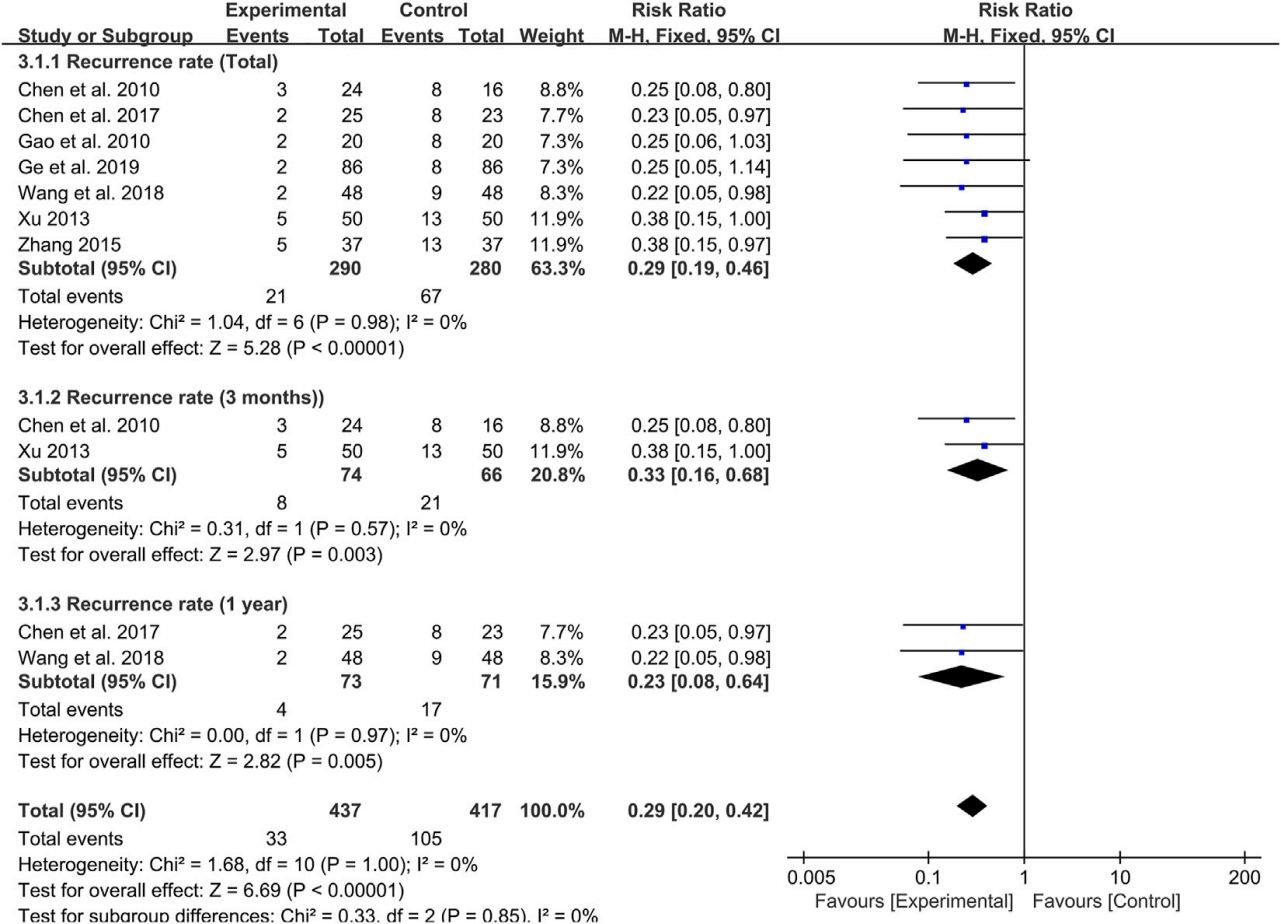
Forest plot displaying the results of the meta-analysis for recurrence rate.

Seven studies were followed up for 8 weeks to 1 year at the end of treatment. Meta-analysis results revealed that probiotics combined with TCM could significantly reduce the recurrence rate of UC patients compared with the control group (RR: 0.29; 95% CI: 0.19, 0.46; *p* < 0.00001, I^2^ = 0%) (Figure 6).

By setting up subgroup analysis, we found that the recurrence rate of patients in the probiotic combined with TCM group were lower than those in the control group at 3 months (RR: 0.33; 95% CI: 0.16, 0.68; *p* = 0.003, I^2^ = 0%) (Figure 6) and 1 year (RR: 0.23; 95% CI: 0.08, 0.64; *p* = 0.005, I^2^ = 0%) (Figure 6) after the end of the course of treatment.

### Meta-analysis of inflammatory cytokines and protein (CRP, TNF-α, IL-6, IL-8 and IL-10)

**FIGURE 7:**
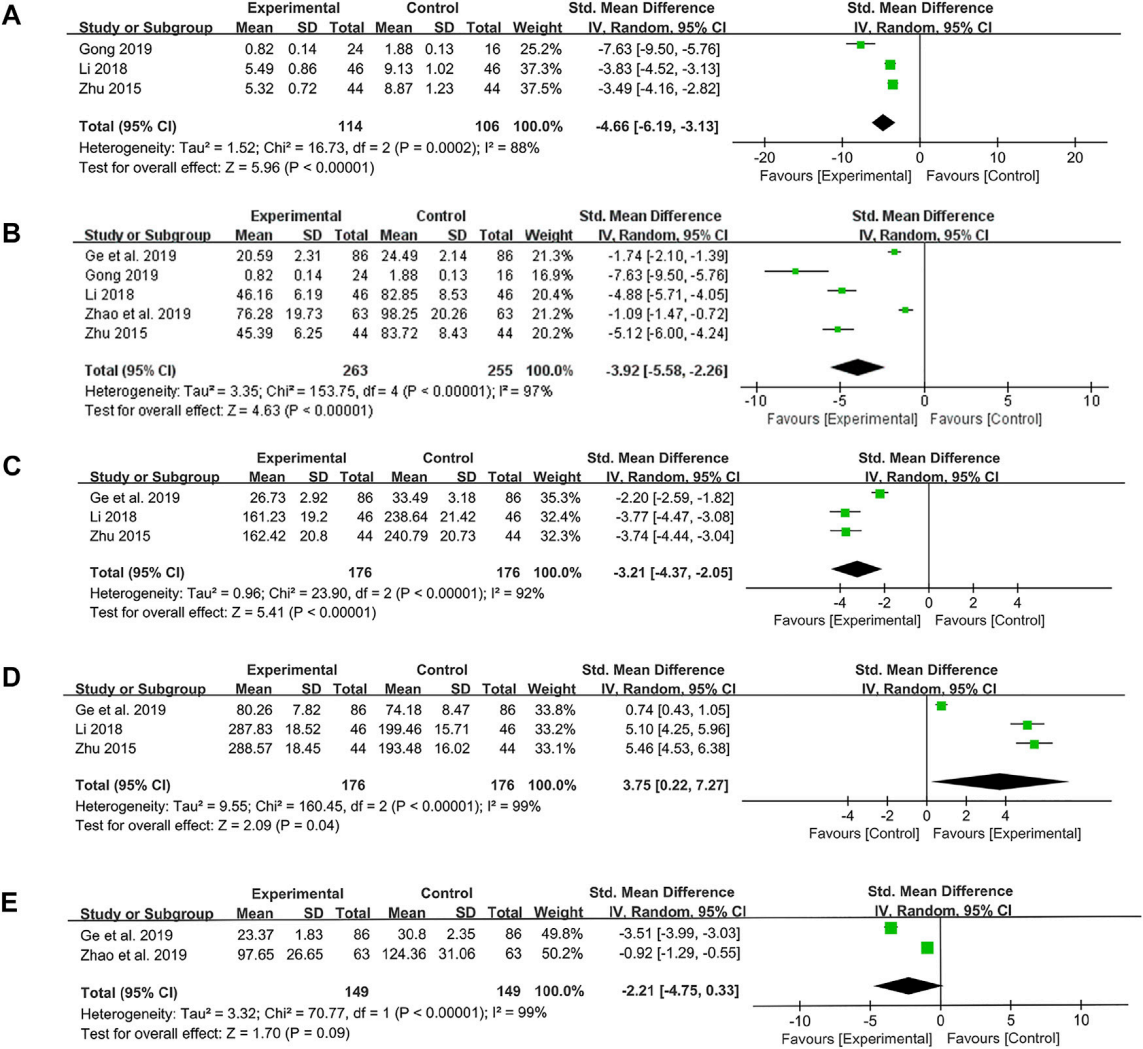
Forest plot displaying the results of the meta-analysis for inflammation-related proteins and cytokines. **(A)** The results of CRP. **(B)** The results of TNF-α. **(C)** The results of IL-8. **(D)** The results of IL-10. **(E)** The results of IL-6.

Three studies were conducted to determine CRP levels. The results showed that probiotics combined with TCM treatment could reduce CRP levels compared with the control group (SMD: −4.66; 95% CI: −6.19, −3.13; *p* < 0.00001, I^2^ = 88%) (Figure 7A).

Five studies were conducted to assess TNF-α level. Similarly, the results of meta-analysis suggested that probiotics combined with TCM could significantly reduce TNF-α level (SMD: −3.92; 95% CI: −5.58, −2.26; *p* < 0.00001, I^2^ = 97%) (Figure 7B).

The same three studies were used to measure the levels of IL-8 and IL-10. We found the probiotics combined with TCM were effective in reducing the level of pro-inflammatory cytokine IL-8 (SMD: −3.21; 95% CI: −4.37, −2.05; *p* < 0.00001, I^2^ = 92%) (Figure 7C) and increasing the level of anti-inflammatory cytokine IL-10 (SMD: 3.75; 95% CI: 0.22, 7.27; *p* = 0.04, I^2^ = 99%) (Figure 7D). Nevertheless, there was no difference in IL-6 levels between probiotics combined with TCM group and control group, which was also a pro-inflammatory cytokine (SMD: −2.21; 95% CI: −4.75, 0.33; *p* = 0.09, I^2^ = 99%) (Figure 7E).

### Meta-Analysis of Adverse Events

**FIGURE 8:**
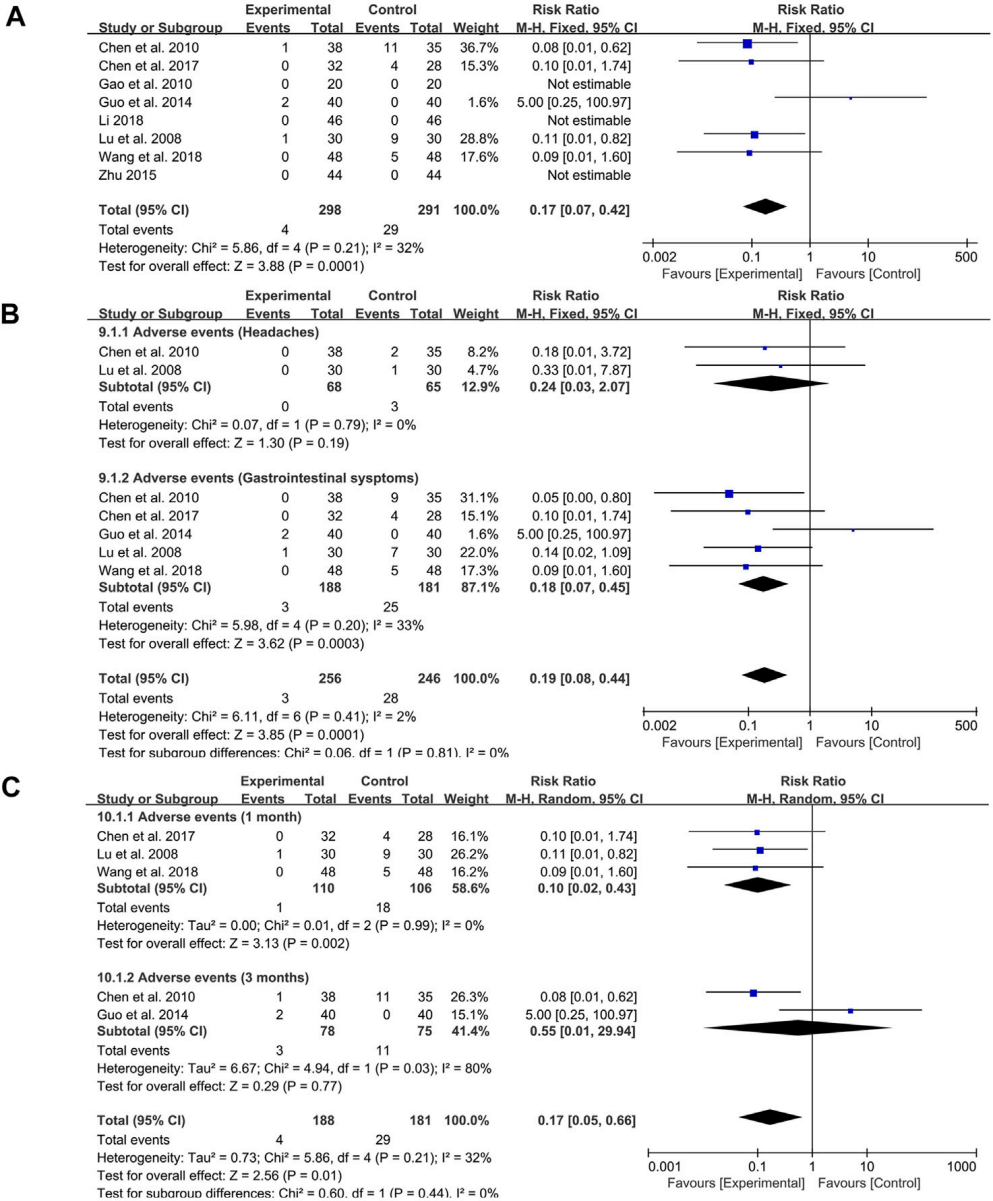
Forest plot displaying the results of the meta-analysis for adverse events. **(A)** The total incidence of adverse events. **(B)** The incidence of specific symptoms of adverse events. **(C)** The incidence of adverse events with different course of treatment.

Among the 15 studies included, five studies reported the occurrence of adverse events, and three studies mentioned that no adverse events occurred in probiotics combined with TCM group and control group. The fixed-effect meta-analysis showed that probiotics combined with TCM could significantly reduce the incidence of adverse events (RR: 0.17; 95% CI: 0.07, 0.42; *p* = 0.0001, I^2^ = 32%) (Figure 8A).

The subgroup analysis indicated that probiotics combined with TCM could significantly reduce the occurrence of gastrointestinal symptoms (RR: 0.18; 95% CI: 0.07, 0.45; *p* = 0.0003, I^2^ = 33%) (Figure 8B) but had no effect on headache (RR: 0.24; 95% CI: 0.03, 2.07; *p* = 0.19, I^2^ = 0%) (Figure 8B).

In addition, due to the different course of treatment, another subgroup analysis was set up. During the course of 1 month, probiotics combined with TCM had obvious effect on reducing the incidence of adverse events (RR: 0.10; 95% CI: 0.02, 0.43; *p* = 0.002, I^2^ = 0%) (Figure 8C). However, there was no significant difference in the occurrence of adverse events between the two groups at 3 months of course of disease (RR: 0.55; 95% CI: 0.01, 29.94; *p* = 0.77, I^2^ = 80%) (Figure 8C).

### Publication Bias

Publication bias was assessed by funnel plots for the included 15 studies which reported the primary outcome. The funnel plots were asymmetrical, suggesting a possible publication bias (Supplementary Figure S1).

### The mechanisms of action of probiotics and TCM in the treatment of UC

#### Mechanism of Action of Probiotics

The dysbiosis of intestinal microbiota in UC patients is characterized by decreased abundance of beneficial bacteria, increased pathogenic bacteria and opportunistic pathogens (Khan et al., 2019). As a result, the treatment strategy for restoring microbial dysbiosis is crucial to the management of UC. Probiotics remain active condition and exhibit beneficial effects on host health. However, probiotics only colonize human intestine for a short time, and did not remain in a broad period of time (Barra et al., 2020). Therefore, the intake of probiotics *in vitro* does not directly colonize the host intestine as beneficial bacteria, but regulates the intestinal microbiota *via* multiple mechanisms to exert therapeutic effects on UC. Probiotics can adhere to epithelial cells during colonization and compete directly with pathogenic bacteria to inhibit their invasion and proliferation. In addition, probiotics can stimulate the production of nutrients needed by beneficial bacteria, such as short-chain fatty acids, thus helping to restore the homeostasis of commensal beneficial bacteria and whole microorganisms in the intestinal tract (María Remes-Troche et al., 2020; Zhang et al., 2021). Mucus barrier and epithelial barrier are important defense lines for host against pathogenic microorganisms as well as harmful substances. Probiotics can stimulate mucus production, repair the mucus barrier, and protect intestine from harmful microorganisms (Glassner et al., 2020). *Faecalibacterium prausnitzii* has been proved to be able to restore the tight junctions of epithelial cells injured by dextran sulfate sodium (DSS) in mice (Carlsson et al., 2013). *Bifidobacterium infantis* can repair epithelial cells and goblet cells damaged by 2,4,6-trinitrobenzene sulfonic acid (TNBS) in rats (Javed et al., 2016), indicating that probiotics have a restorative effect on epithelial barrier. Moreover, probiotics also have the potential to reduce host intestinal permeability (Darb Emamie et al., 2021).The recovery and improvement of intestinal barrier function prevents the interaction between intestinal lumen and pathogenic microorganisms, and further reduces the immune response and inflammation response in the intestine, indicating the indirect anti-inflammatory effect of probiotics. Indeed, probiotics can also reduce inflammation and regulate the immune system directly. It is proved that *lactobacillus acidophilus*, *bifidobacterium bifidum*, and *streptococcus* can decrease the expression of pro-inflammatory cytokine IL-1β and increase the expression of anti-inflammatory cytokine IL-10 in UC patients (Li et al., 2012).

Furthermore, anti-inflammatory cytokines such as IL-10, IL-27 and IL-35 can be expressed and produced by *Lactobacillus* and non-pathogenic *Escherichia coli* (Barra et al., 2020). *Saccharomyces boulardii* can ameliorate DSS-induced colitis in mice by inhibiting the activation of nuclear factor kappa-B (NF-κB) signaling pathway and inhibiting the release of pro-inflammatory cytokines (Gao et al., 2021). Notably, increased short-chain fatty acids generation in response to probiotics can decrease TNF-α and IL-12, increase IL-10, inhibit NF-κB signaling pathway, and produce anti-inflammatory effects (Säemann et al., 2000; Segain, 2000).

Probiotics can regulate the balance of immune cells in addition to inhibiting the inflammatory response. Th17 cells are associated with the pathogenesis of UC and Crohn’s disease, and an imbalance of Th17/Treg cells is a prominent contributor of decreased host tolerance to intestinal microorganisms and inflammatory responses (Eastaff-Leung et al., 2010). The polysaccharide produced by *Bacteroides fragilis* can decrease the expression of IL-17 to reduce the differentiation of Th17 cells (Mazmanian et al., 2008). Another probiotic, *Clostridium* can induce Treg cell production *via* its metabolites (Atarashi et al., 2011). In summary, exogenous probiotics can repair intestinal microbiota disorder, restore intestinal mucus and epithelial barrier, and inhibit abnormal immune response, which is the primary mechanisms of probiotics in the treatment of UC. Moreover, since different strains have distinct metabolic activities and mechanisms of action, various probiotic products may exert their therapeutic benefits through multiple pathways, indicating that multi-strain combinations may be more effective than simple probiotic (Oka and Sartor, 2020).

#### Mechanism of Action of TCM

UC is an idiopathic inflammatory disease in essence. Abnormal mucosal inflammation is a key basis for the existence and progress of UC. TCM made of natural materials is considered superior to first-line clinical drugs for UC with fewer side effects (Khan et al., 2019). TCM has immunomodulatory and anti-inflammatory effects in treating UC (Sałaga et al., 2014; Xiong et al., 2021). Curcumin, the main active ingredient of the dried rhizome of *Curcuma longa* L. (*jiāng huáng*), has attracted much attention due to its potent anti-inflammatory effects. Curcumin can inhibit the activation of NF-κB signaling pathway and alleviate intestinal inflammation by inhibiting the levels of IL-1, IL-2 and TNF-α (Mazieiro et al., 2018). NF-κB pathway plays a key role in the release of intestinal inflammatory cytokines and participated in the intestinal inflammation and immune response in UC patients (Lu and Zhao, 2020). The dried processed product of leaf or stem and leaf of *Strobilanthes cusia* (Nees) Kuntze (*qīng dài*), the dried rhizome of *Scutellaria baicalensis* Georgi (*huáng qín*), and the dried aboveground part of *Andrographis paniculata* (Burm.f.) Nees (*chuān xīn lián*), which are commonly used in the treatment of UC, have been demonstrated to have the effect on the inhibition of the NF-κB signaling pathway (Gao et al., 2016, 2018; Liu et al., 2020; Sun et al., 2020). In addition to NF-κB pathway, TCM can also regulate mitogen-activated protein kinase (MAPK), NOD-like receptor thermal protein domain associated protein 3 (NLRP3) inflammasome, and other signaling pathways involved in the inflammatory process of UC (Ma et al., 2019; Chao et al., 2020; Hu J. et al., 2021). Alkaloids, phenols, flavonoids and other ingredients found in herbal medicinals, have significant anti-inflammatory effects, which may be the main source of the anti-inflammatory activity of TCM in the treatment of UC (Cao et al., 2019). Notably, Rhubarb and peony Decoction (*Dà Huáng Mŭ Dān Tāng*) can regulate the balance of Th17/Treg cells to improve UC. Another formula, Scutellaria Decoction (*Huáng Qín Tāng*) can restore the drift of Th1/Th17 cells and the proliferation of Treg cells to improve experimental colitis, which also indicate the advantages and potential of TCM in immune regulation (Zou et al., 2015; Luo et al., 2019).

Most studies have begun to focus on the influences of TCM on the intestinal microbial dysbiosis in UC. Rhein, the active ingredient of the dried root and rhizome of *Rheum palmatum* L. (*dà huáng*), has therapeutic effect on experimental colitis by restoring intestinal microbial dysbiosis (Wu et al., 2020). Moreover, *qīng dài* and *huáng qín*, as well as formulas such as Ginseng, Poria and Atractylodes Macrocephalae Powder (*Shēn Líng Bái Zhú Săn*), Coptis Toxin-Resolving Decoction (*Huáng Lián Jiĕ Dú Tāng*), and Stomach-Calming Powder (*Píng Wèi Săn*), can inhibit experimental colitis by restoring microbial diversity, increasing the abundance of beneficial bacteria, and inhibiting the growth of pathogenic bacteria (Ma et al., 2019; Zhang Z. et al., 2020; Qi-yue et al., 2020; Yuan et al., 2020; Zhu et al., 2020). Short-chain fatty acids produced by intestinal microbiota are not only nutrients for microorganisms and immune regulation participants, but also the major energy source of colonic epithelial cells, and their production helps to enhance the function of the epithelial barrier (Parada Venegas et al., 2019). TCM can promote the production of short-chain fatty acids by increasing the abundance of short-chain fatty acid-producing bacteria, thereby further regulating the intestinal immune system and restoring the epithelial barrier (Yuan et al., 2020; Zhu et al., 2020). Notably, TCM and its active compounds can directly restore the intestinal epithelial barrier by reducing mucosal damage, alleviating crypt degeneration, and promoting the expression of intestinal tight junction protein, which is also an important manifestation of the multi-component and multi-target mechanism of TCM on UC (Gao et al., 2016; Cao et al., 2019; Chao et al., 2020).

#### The Interaction of Probiotics and TCM

Polysaccharides contained in various TCM not only have immune-regulatory effect, but also can be used as prebiotics to promote the growth of probiotics (Foley et al., 2016). Prebiotics are “substrates selectively used by host microorganisms for health benefits” (Gibson et al., 2017). Prebiotics can produce substances which are beneficial to the growth of beneficial bacteria through the metabolism of probiotics and intestinal commensal bacteria (Damaskos and Kolios, 2008). *Lactobacillus* and *Bifidobacterium* are two most widely used probiotic strains currently, and their abundance in host intestine can be improved by polysaccharides extracted from the dried root of *Astragalus mongholicus* Bunge (*huáng qí*) and the dried aboveground part of *Portulaca oleracea* L. (*mă chĭ xiàn*). The extracts of the dried root of *Codonopsis pilosula* (Franch.) Nannf. (*dăng shēn*), the dried root and rhizome of steamed *Panax ginseng* C.A.Mey. (*hóng shēn*) and the dried ripe kernel of *Coix lacryma-jobi* L. (*yì yĭ rén*) can also increase the abundance of *Lactobacillus* and *Bifidobacterium*, while *dăng shēn* can also promote the abundance of another probiotic, *A. muciniphila*, further inhibit the growth of pathogenic bacteria and alleviate colonic inflammation in mice (Guo et al., 2015; Jing et al., 2018). In addition, salvianolic acid A, the main active ingredient of the dried root and rhizome of *Salvia miltiorrhiza* Bunge (*dān shēn*), can also regulate epithelial cell tight junction protein and promote the repair of epithelial barrier by increasing the abundance of *A. muciniphila* (Wang K. et al., 2018). These findings suggest that TCM can be used as potential prebiotics in combination with probiotics, enhance the activity and abundance of probiotics, and further lead to synergistic effect on the treatment of UC. Probiotics can also play a role in enhancing the efficacy of TCM in combination. Many active ingredients in TCM are macromolecular substances with limited oral bioavailability, which are difficult to be absorbed by the body. However, probiotics help to further decompose the huge molecules of TCM (Su et al., 2021). After fermentation by *Lactobacillus rhamnosus* and *Bacillus natto,* fermented *dān shēn* can promote proliferation and differentiation of intestinal epithelial cells and regulate immunity. The alleviative effect of fermented *dān shēn* on DSS-induced colitis is better than that of unfermented *dān shēn*. In addition, the fermentation process facilitated the decomposition of macromolecular substances in *dān shēn* which was difficult to be absorbed by the body (Su et al., 2021). At the same time, another study found that probiotic fermented the dried rhizome of *Atractylodes macrocephala* Koidz. (*bái zhú*) had better protective effects on intestinal epithelial cells damaged by lipopolysaccharide (Bose and Kim, 2013).

Ginsenosides are important active ingredients of the dried root and rhizome of *Panax ginseng* C.A.Mey. (*rén shēn*). Some probiotic bacteria, such as *Bifidobacterium* and *Bacillus*, can convert protopanaxadiol ginsenosides Rg3 and Rg5 into ginsenosides Rh2 and Rh3, and promote the partial conversation of protopanaxadiol ginsenosides Rb1, Rb2, and Rc cleavage sugars into monoglycosylated ginsenoside compound K, which ultimately improves the anti-inflammatory and antitumor activities of ginseng (Kim, 2018). Thus, probiotics will be more conducive to improving the bioavailability of the active ingredients of TCM, transforming insoluble polysaccharides into monosaccharides, decomposing macromolecular proteins into polypeptides and amino acids, and deriving unstable compounds into stable substances, so as to improve the efficacy and better therapeutic potential of combined drugs (Wang et al., 2021).

**FIGURE 9:**
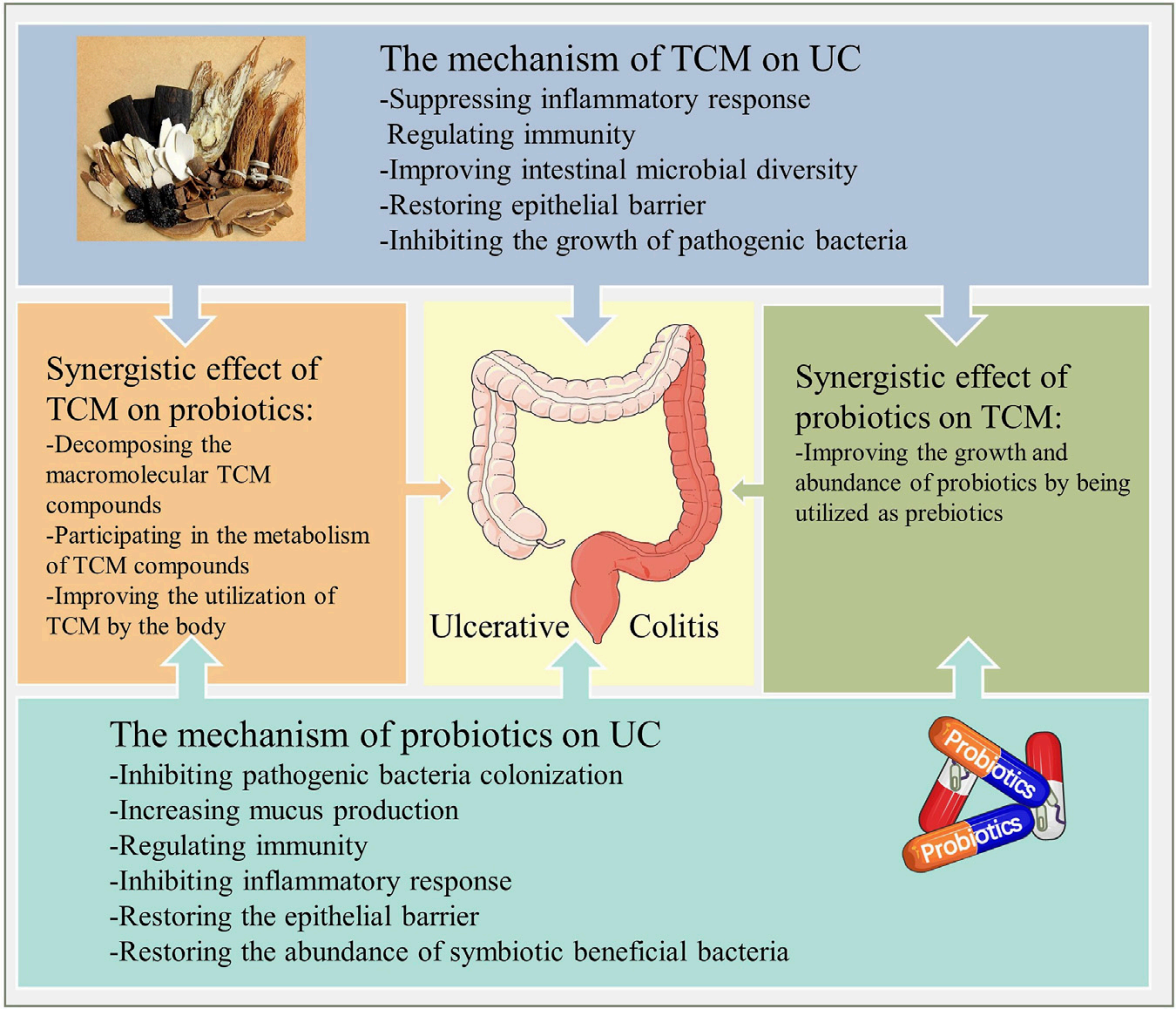
The mechanism of action of probiotics combined with TCM for UC. Probiotics and TCM exert therapeutic effects through their respective actions, while the interaction between them reflects the synergistic effect and necessity of this combination.

In summary, the mutual synergistic effect between probiotics and TCM not only plays a therapeutic role through its unique biological activity but also is an important mechanism leading to the improvement of clinical efficacy (Figure 9).

## Discussion

As a persistent intestinal inflammatory disease, UC has been proved to be an important risk factor for colorectal cancer (Rabbenou and Ullman, 2020). The incidence of colorectal cancer in UC patients is about 2.4 times higher than general population. Such incidence is relevant to the duration of the disease and the severity of intestinal histologic inflammation (Jess et al., 2012; Choi et al., 2019). To date, 5-ASA is the most widely used first-line drug, especially for mild to moderate UC (Kobayashi et al., 2020). However, non-response and intolerance prevent a proportion of patients from receiving 5-ASA treatment, and such patients need to rely on drugs such as corticosteroids and thiopurines (le Berre et al., 2020). Likewise, the significant toxicity of long-term use of corticosteroids and thiopurines has attracted public attention (van Gennep et al., 2018; Salice et al., 2019). It is urgent to explore more efficient and safe treatment strategies for UC.

The therapeutic strategies targeting the host intestinal microbiota have been suggested for the treatment of ulcerative colitis (Hu Y. et al., 2021). Probiotics is considered to be a promising treatment approaches in recent years. A tremendous number of functional foods containing probiotics are currently accessible, indicating that probiotics appear to be quite safe.

The probiotics products *Escherichia coli* Nissle 1917, as well as VSL#3 (a food product including *L. paracasei*, *L. plantarum*, *L. acidophilus*, *L. delbrueckii*, *B. longum*, *B. breve*, *B. infantis*, and *Streptococcus thermophilus*), have been proved to have therapeutic effects on UC (Chen et al., 2020). Although numerous RCTs have reported the efficacy of probiotics, some researchers still have found no significant difference in efficacy between probiotics and placebo (Groeger et al., 2013; Matsuoka et al., 2018). Similarly, various systematic studies have been conducted to evaluate the therapeutic potential of probiotics. Several studies have supported the effectiveness of probiotics in induction therapy, but have also found its poor efficacy in maintenance therapy (Losurdo et al., 2015; Chen M. et al., 2021). Therefore, the application of probiotics alone as a formal clinical treatment of UC may be insufficient. The efficacy of probiotics combined with TCM on non-alcoholic fatty liver disease, type 2 diabetes and other diseases has been explored (Zhang Y. et al., 2020; Wang et al., 2020). In preclinical studies, TCM has also proved its therapeutic effect on UC by regulating the intestinal microbiota and restoring intestinal microbial dysbiosis (Chen K. et al., 2021). Besides, TCM can act as prebiotics to promote the activity and improve the efficacy of probiotics (Chang et al., 2015). In China, the combination of probiotics and TCM has been used in the clinical practice of UC. Apparently, a systematic evaluation of the combined application of probiotics and TCM can not only assess the efficacy and safety, but also may contribute to a new clinical protocol in the treatment of UC. 14 RCTs containing 1,154 patients were included in this meta-analysis. The results of our meta-analysis showed that the clinical effective rate of probiotics combined with TCM was more remarkable than that of the control group. In fact, 5-ASA can be used as the preferred drug for maintenance treatment of mild to moderate UC and the adjuvant drug for moderate to severe UC, which is beneficial to the majority of UC patients. Long-term use of 5-ASA reduces the risk of relapse and cancer in UC patients, making it an indispensable drug for UC treatment (Kim and Kim, 2010; Wang C.-S.-E. et al., 2018). Remarkably, our subgroup data revealed that probiotics combined with TCM are more effective than 5-ASA for UC, which means that this treatment strategy is likely to beneficial to UC patients who lack response or are intolerant to 5-ASA treatment, so as to avoid the use of corticosteroids and thiopurines with more obvious toxic side effects. Furthermore, a considerable number of RCTs have assessed the efficacy of probiotics or TCM in the treatment of UC, with a range of studies supporting their therapeutic efficacy on their own. Our subgroup analysis showed that the efficacy of probiotics combined with TCM in the treatment of UC was better than that of probiotics or TCM alone, implying that there may be a synergistic effect between the two to improve the efficacy, and also provides a new strategy and concept for the treatment of UC.

The establishment of a treatment course is essential for a treatment strategy. Therefore, by establishing a subgroup analysis with different treatment regimens, we found that probiotics combined with TCM had significant therapeutic benefits for UC at both 1 month and 3 months of continuous treatment. However, in the analysis of five studies with 2 months as the treatment course, the results showed that there was no significant difference between the effectiveness of probiotics combined with TCM and the control measures. The reason for this result may be due to the limited number of studies included in the analysis. Nevertheless, this phenomenon indicates that the treatment course of probiotics and TCM application can have a certain impact on the clinical efficiency. Moreover, due to the lack of long-term treatment process in the studies included in this analysis, more clinical studies are still needed to evaluate the safety of probiotics and TCM in long-term application.

Furthermore, the recurrence rate showed the combination of probiotics and TCM can effectively control the disease process and slow down the recurrence of UC. Abnormal immune response is considered to be the key to the pathogenesis of UC. Cytokines regulated and released by multiple inflammatory signaling pathways are the central component of active or chronic inflammation in UC (Bamias et al., 2011). IL-6 induces the recruitment and activation of macrophages in the intestinal epithelium (Bamias et al., 2012), and activated macrophages can further produce excessive IL-6 and TNF-α by phagocytosis of microbiota (Chang, 2020). In addition, IL-6 can up-regulate the expression of IL-23R, promote IL-23-related reactions, further induce the differentiation of Th17 cells, lead to excessive release of downstream cytokines such as IL-17 and IL-22 and aggravate inflammation. TNF-α can increase inflammatory response by inducing Paneth cells necrosis, damaging epithelial cells, and activating intestinal macrophages (Günther et al., 2011). Therefore, IL-6 and TNF-α are considered to be important mediators of chronic intestinal inflammation (Pugliese et al., 2017; Neurath, 2019). IL-8 is involved in neutrophils recruitment and activation (Kim and Kim, 2010; Lopetuso et al., 2020). When neutrophils are activated, the molecules contained in various intracellular particles are released, resulting in the release of pro-inflammatory cytokines. Therefore, IL-8 is also considered as a key marker of inflammatory activity in response to UC (Brown et al., 2002; Bamias et al., 2012; Rodríguez-Perlvárez et al., 2012). Our meta-analysis results showed that after the application of probiotics and TCM, TNF-α and IL-8 decreased significantly, while IL-6 did not decrease significantly. In fact, TNF-α is released not only by macrophages but also by fibroblasts and T cells (Pugliese et al., 2017). Probiotics combined with TCM have an advantage in reducing IL-8 expression and inhibiting neutrophil activation, but may not significantly regulate IL-6-macrophage- TNF-α release pathway (Pepys and Hirschfield, 2003; Zilberman et al., 2006). After probiotics combined TCM treatment, CRP decreased significantly. Meanwhile, IL-10 acts as an anti-inflammatory cytokine in UC. It was found that the deficiency of IL-10 and its receptor IL-10R was associated with the occurrence of spontaneous colitis (Neurath, 2014). The recovery effect of probiotics combined with TCM on IL-10 also suggests their inhibitory effect on intestinal inflammation. Remarkably, analysis results for all five inflammatory cytokines were highly heterogeneous, which may be due to the use of different units and equipment in the original research measurement, taking into account the wide time range of the original literature. Although we used SMD to avoid the impact of these factors as much as possible, we should still take the findings here with caution.

The incidence of adverse events is an important indicator for evaluating the safety of treatment strategies. Compared with drugs with significant toxic side effects such as corticosteroids and thiopurines, UC patients tend not to experience serious adverse events with 5-ASA (van Gennep et al., 2018). However, some studies have reported that mesalazine may still cause adverse effects such as fever, nausea, dizziness, and diarrhea in UC patients (Sehgal et al., 2018). The results of the present meta-analysis show that probiotics combined with TCM treatment significantly reduce the occurrence of adverse events. Among the five studies that reported adverse events, the control drugs in four studies were all 5-ASA. The original data of all the four studies mentioned that the incidence of adverse events in the in the combination of probiotics and TCM was significantly lower than that of 5-ASA (Lu et al., 2008; Chen et al., 2010, 2017; Wang and Liu, 2018). Although all five studies reported adverse events in both probiotics combined TCM groups and control groups, the specific symptoms were limited to headache and gastrointestinal symptoms. Subgroup analysis showed that probiotics combined with TCM could reduce the occurrence of gastrointestinal symptoms. In addition, we found that probiotics combined with TCM were significantly less likely to cause adverse events than the control group when used for 1 month of treatment in UC patients. However, there was no statistically difference in the adverse events at 3-month course of treatment, and there was a lack of specific data in the included studies for treatment course of more than 3 months. As a result, although probiotics combined with TCM can reduce the occurrence of adverse events in UC patients, there is still a lack of evaluation of the safety of their long-term application.

Since the specific probiotic species used in the included studies were different, we not only set up a subgroup analysis to evaluate whether the use of different probiotics affects clinical effective rate, but also combed and analyzed the frequency of probiotics use (Table 1). Of all the included original studies, the three most commonly used probiotics were *Enterococcus Faecium*, *Lactobacillus acidophilus* and *Bifidobacterium infantis*, with frequencies of 10, 8 and 7, respectively. Secondly, Bacillus *cereus*, *Bacillus licheniformis* and *Bacillus subtilis* all had frequencies of 3. Finally, both *Lactobacillus lactis* and *Lactococcus lactis subsp. Lactis* were each used only once. Experts believe that mixtures of different strains may be more effective than a single strain due to the different metabolic characteristics and immunomodulatory activities of various probiotics (Oka and Sartor, 2020). Our meta-analysis also confirm that the mixed use of multiple probiotics may be more advantageous. Cocktail therapy is a therapeutic strategy, which combines a variety of drugs through different pharmacological pathways to play a therapeutic effect. This strategy has advantages in avoiding drug resistance and improving drug efficacy. Cocktail therapy is effective for the treatment of a variety of serious illnesses, including acquired immunodeficiency syndrome (AIDS) and cancer. Based on the results of subgroup analysis, the combination of different probiotic species improves the clinical therapeutic effect, such “cocktail therapy” may also be a potential strategy for the treatment of UC.

In terms of the type of probiotic used, 10 of the 14 included studies used *Enterococcus Faecium*. *Lactobacillus acidophilus* and *Bifidobacterium infantis* were also the two strains with high frequently of use. In all included studies, the combined use of these three probiotics was the most common. In fact, a retrospective study has found that *Enterococcus Faecium*, *Lactobacillus acidophilus* and *Bifidobacterium infantis* are the most commonly used probiotic strains in the treatment of IBD. The mechanisms may be based on inhibiting pathogenic colonization, producing beneficial products through metabolic activity and promoting intestinal mucus (Amer et al., 2018; He et al., 2019). The combination of *Enterococcus faecium*, *Lactobacillus acidophilus*, *Bifidobacterium infantis*, and *Bacillus cereus* has also been used in the original studies. However, it is worth noting that some studies support the potential threat of *Bacillus cereus* opportunistic foodborne pathogenic bacteria to human health (Zhu et al., 2016; Cui et al., 2019). Therefore, the safety of multiple probiotic strains for long-term use, especially *Enterococcus Faecium*, *Lactobacillus acidophilus*, *Bifidobacterium infantis*, and *Bacillus cereus*, still is an urgent need for more in-depth evaluation of the corresponding research.

To determine the characteristics and patterns of specific herbal medicinals used in the included studies (the details of medicinals and dosages used in original studies could be seen in Supplementary Table S1), we analyzed the frequency of herbal medicinals used in each original study. The high-frequency medicinals (frequency ≥3) are the dried root and rhizome of *Glycyrrhiza glabra* L. (*gān căo*), the dried rhizome of *Atractylodes macrocephala* Koidz. (*bái zhú*), the dried rhizome of *Coptis chinensis* Franch. (*huáng lián*), the dried root of Astragalus mongholicus Bunge (*huáng qí*), the dried ripe seed of *Dolichos lablab* L. (*bái biăn dòu*), the dried root of *Paeonia lactiflora* Pall. (*bái sháo*), the dried sclerotium of *Poria cocos* (Schw.) Wolf *(fú líng*), the dried root of *Pulsatilla chinensis* (Bunge) Regel (*bái tóu wēng*), the dried root and rhizome of *Salvia miltiorrhiza* Bunge (*dān shēn*), the dried root of *Codonopsis pilosula* (Franch.) Nannf. (*dăng shēn*), the dried processed product of leaf or stem and leaf of *Strobilanthes cusia* (Nees) Kuntze (*qīng dài*) and the dried rhizome of *Dioscorea oppositifolia* L. (*shān yào*), with frequencies of 8, 6, 5, 5, 4, 4, 4, 3, 3, 3, 3, 3, respectively. We further divided these high-frequency medicinals into categories based on their effects. The dried root and rhizome of *Glycyrrhiza glabra* L. (*gān căo*), *bái zhú*, *dăng shēn*, *huáng qí*, the dried ripe seed of *Dolichos lablab* L. (*bái biăn dòu*), the dried sclerotium of *Poria cocos* (Schw.) Wolf (*fú líng*) and the dried rhizome of *Dioscorea oppositifolia* L. (*shān yào*) are all Qi-boosting and spleen-strengthening medicinals, which are suitable for UC patients with spleen deficiency and dampness accumulation.

The dried rhizome of *Coptis chinensis* Franch. (*huáng lián*), the dried root of *Pulsatilla chinensis* (Bunge) Regel (*bái tóu wēng*), *dān shēn*, the dried root of *Paeonia lactiflora* Pall. (*bái sháo*) and *qīng dài* are suitable for UC patients with damp-heat syndrome in the large intestine, which have the effects of clearing heat, drying dampness, cooling blood, and detoxication. TCM is characterized by the treatment based on syndrome differentiation (Xie et al., 2020). According to the relevant study, the spleen deficiency and dampness syndrome and large intestine damp-heat syndrome are the two most common syndromes of UC patients (Zhang et al., 2019). Consequently, medicinals that with effects of qi-boosting, spleen-strengthening, heat-clearing, dampness-drying, blood-cooling, and detoxication are frequently utilized in the treatment of UC patients, and a study on the medication pattern of ulcerative colitis in China supports this conclusion (Jia et al., 2021).

There are still several limits in our study. Firstly, the number of original studies included in this meta-analysis was limited, and some outcomes, such as CRP, IL-8, and IL-10, were not recorded in sufficient trials, potentially biasing the results of meta-analysis. Secondly, the lack of a substantial number of high-quality, multicenter standard RCTs in the included studies, as well as the lack of explanations of random assignment procedures and blinding in some studies, may be the risk factors affecting the quality of our meta-analysis. Moreover, despite using SMD to reduce the impact of diverse data measurement units and methods on the results, the results of the inflammatory cytokines in this meta-analysis still had a high level of heterogeneity. At last, due to a lack of necessary data, we could not comprehensively evaluate the long-term efficacy and safety of probiotics combined with TCM. It is hoped that more relevant studies will be conducted in the future to promote the rational use of probiotics combined with TCM in the treatment of UC.

## Conclusion

Probiotics combined with TCM can improve the clinical efficiency of UC patients, reduce the recurrence rate and adverse events, suggesting that the combination has the potential for the treatment of UC and has significant clinical promotion value. The interaction between probiotics and TCM improve their therapeutic effects on UC. The combination of different probiotic with TCM guided by syndrome differentiation is a valuable protocol to increase clinical efficacy. More high-quality RCTs are still needed to evaluate the long-term efficacy and safety of probiotics combined with TCM in the treatment of UC.

## Data Availability

The original contributions presented in the study are included in the article/Supplementary Material, further inquiries can be directed to the corresponding author.

## Abbreviations

AIDS, Acquired immunodeficiency syndrome; 5-ASA, 5-aminosalicylic acid; CBM, Chinese biomedical literature service system; CI, Confidence interval; CNKI, China Academic Journals; CQVIP, Chinese Science and Technology Journals; CRP, C-reaction protein; DSS, Dextran sulfate sodium; IBD, Inflammatory bowel disease; IL, Interleukin; MAPK, Mitogen-activated protein kinase; NF-κB, Nuclear factor kappa-B; NLRP3, NOD-like receptor thermal protein domain associated protein 3; RCTs, Randomized controlled trials; RR, Risk ratio; SMD, standardized mean difference; TCM, Traditional Chinese medicine; TNBS, 2,4,6-trinitrobenzene sulfonic acid; TNF-α, Tumor necrosis factor-α; UC, Ulcerative colitis.
