# Supplementary material for: Efficacy and Safety of Probiotics Combined With Traditional Chinese Medicine for Ulcerative Colitis: A Systematic Review and Meta-Analysis
Source: Front Pharmacol. 2022 Mar 7;13:844961. doi: 10.3389/fphar.2022.844961 (PMC8936956; doi:10.3389/fphar.2022.844961)
Supplement: Supplementary file 1 [file DataSheet1.PDF]

1 Supplementary Figure

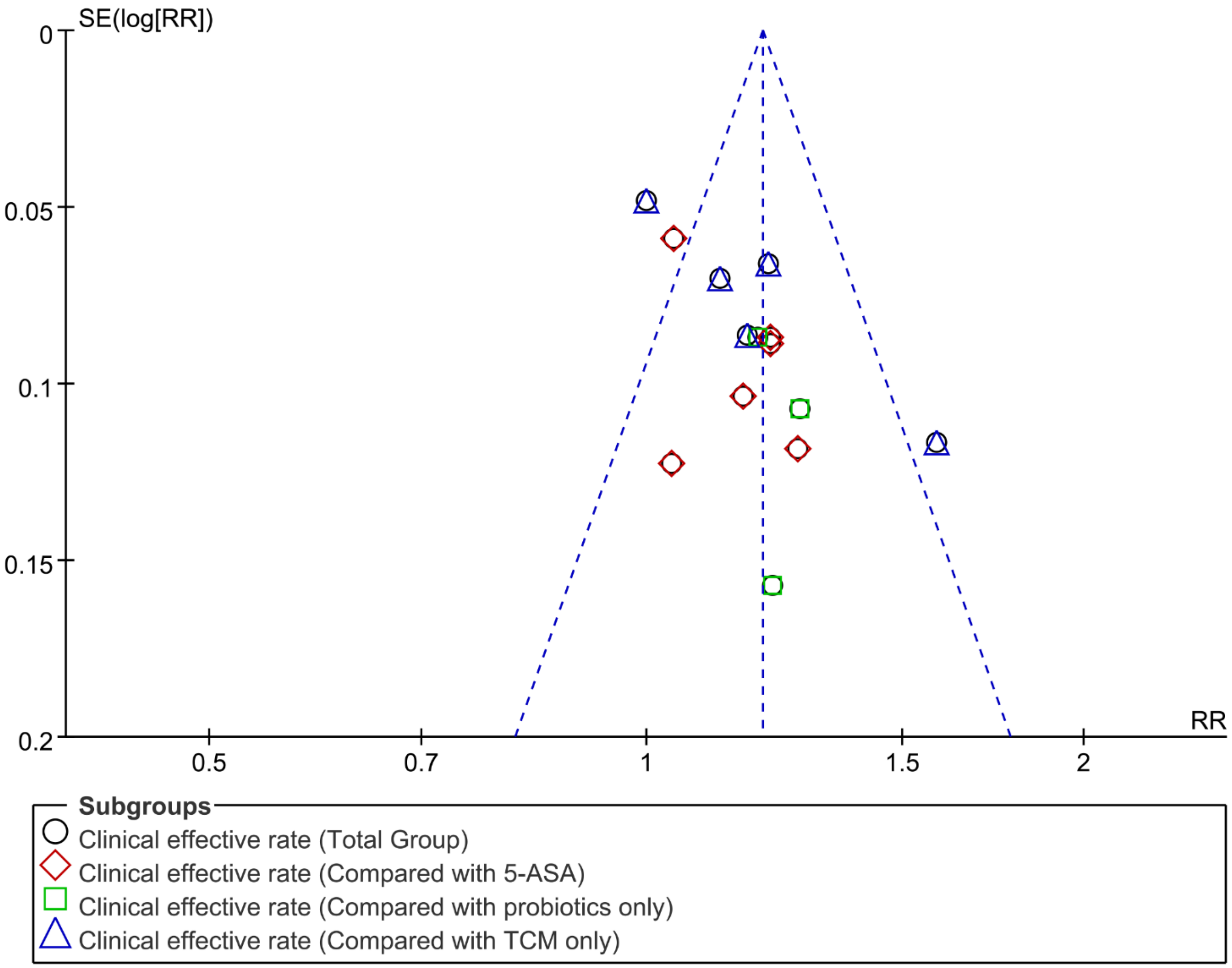

Supplementary Figure 1. Funnel plot to assess publication bias for primary outcome in this meta-analysis..

2 Supplementary Table

Supplementary Table 1: The medicinals and dosages used in the original studies

Study	Formulas	Medicinals and dosages	Source
Gong 2019	Radix Puerariae Lobatae, Radix Scutellariae and Rhizoma Coptidis Decoction (*Gé Gēn Qín Lián Tāng*)	The dried root of *Pueraria lobata* (Willd.) Ohwi (*gé gēn*), 30g; the dried root of *Scutellaria baicalensis* Georgi (*huáng qín*), 15g; the dried rhizome of *Coptis chinensis* Franch. (*huáng lián*), 10g; the dried root and rhizome of *Glycyrrhiza glabra* L. (*gān cǎo*), 15g.	Prepared by Gong.
Ge et al. 2019	Astragalus Granule (*Huáng Qí Kē Lì*)	The dried root of *Astragalus mongholicus* Bunge (*huáng qí*), 12g	Sichuan Baili Pharmaceutical Co., Ltd., China.
Zhao et al. 2019	Radix Scutellariae Decoction (*Huáng Qín Tāng*)	The dried root of *Scutellaria baicalensis* Georgi (*huáng qín*), 20g; the dried root and rhizome of *Glycyrrhiza glabra* L. (*gān cǎo*), 6g; the dried root of *Paeonia lactiflora* Pall. (*bái sháo*), 10g; the dried ripe fruit of *Ziziphus jujuba* Mill. (*dà zǎo*), 10g.	Guangdong Kang'erdan Pharmaceutical Co., Ltd., China.
Wang et al. 2018	Compound Indigo Naturalis Granule (*Fù Fāng Qīng Dài Kē Lì*)	The dried processed product of leaf or stem and leaf of *Strobilanthes cusia* (Nees) Kuntze (*qīng dài*); the dried aboveground part of *Portulaca oleracea* L. (*mǎ chǐ xiàn*); the dried root of *Angelica dahurica* (Hoffm.) Benth. & Hook.f. ex Franch. & Sav. (*bái zhǐ*); the dried root of Smilax glabra Roxb. (*tǔ fú líng*); the dried root of *Arnebia euchroma* (Royle ex Benth.) I.M.Johnst. (*zǐ cǎo*); the dried rhizome or petiole residues of *Dryopteris crassirhizoma* Nakai (*mián mǎ guàn zhòng*); the dried herb of *Taraxacum mongolicum* Hand.-Mazz.(*pú gōng yīng*); the dried root and rhizome of *Salvia miltiorrhiza* Bunge (*dān shēn*); the dried rhizome of	Shaanxi Pharmaceutical Holding Group Tianning Pharmaceutical Co., Ltd., China.

		Dioscorea collettii var. hypoglauca (Palib.) S.J.Pei & C.T.Ting (*bì xiè*); the dried velamen of *Dictamnus dasycarpus* Turcz.(*bái xiān pí*); the dried fruit of *Prunus mume* (Siebold) Siebold & Zucc. (*wū méi*); the dried ripe fruit of *Crataegus pinnatifida* Bunge (*shān zhā*); the dried ripe fruit of *Schisandra chinensis* (Turcz.) Baill. (*wŭ wèi zĭ*); *shén qū*.	
Li 2018	Spleen-strengthening<br>And Dampness-drying Decoction (*Jiàn Pí Lì Shī Tāng*)	The dried rhizome of *Atractylodes macrocephala* Koidz. (*bái zhú*), 15g; the dried ripe seed of Dolichos lablab L. (*bái biăn dòu*), 10g; the dried rhizome of *Dioscorea oppositifolia* L. (*shān yào*), 18g; the dried root of *Pulsatilla chinensis* (Bunge) Regel (*bái tóu wēng*), 12g; the dried root and rhizome of *Glycyrrhiza glabra* L. (*gān căo*), 6g; the dried root of *Codonopsis pilosula* (Franch.) Nannf. (*dăng shēn*), 15g; the dried root of *Astragalus mongholicus* Bunge (*huáng qí*), 18g; the dried root of *Paeonia lactiflora* Pall. (*bái sháo*), 10g; the dried rhizome of *Coptis chinensis* Franch. (*huáng lián*), 6g; the dried sclerotium of *Poria cocos* (Schw.) Wolf (*fú líng*), 15g.	Prepared by Li.
Chen et al. 2017	Compound Indigo Naturalis Granule (*Fù Fāng Qīng Dài Kē Lì*)	The dried processed product of leaf or stem and leaf of *Strobilanthes cusia* (Nees) Kuntze (*qīng dài*), 30g; the calcined products of alum (*kū fán*), 6g; the dried condensed decoction of *Senegalia catechu* (L.f.) P.J.H.Hurter & Mabb. (*ér chá*), 15g; the pearl produced by *Pteria martensii* (Dunker) (*zhēn zhū*), 3g; the dried bark of *Phellodendron chinense* C.K.Schneid. (*huáng băi*), 21g.	Prepared by Chen et al.
Zhu 2015	Spleen-strengthening<br>And Dampness-drying	The dried rhizome of *Atractylodes macrocephala* Koidz. (*bái zhú*), 15g; the dried ripe seed of Dolichos lablab L. (*bái biăn dòu*), 10g; the dried rhizome of *Dioscorea oppositifolia* L. (*shān yào*), 18g;	Prepared by Zhu.

	Decoction (*Jiàn Pí Lì Shī Tāng*)	the dried root of *Pulsatilla chinensis* (Bunge) Regel (*bái tóu wēng*), 12g; the dried root and rhizome of *Glycyrrhiza glabra* L. (*gān cǎo*), 6g; the dried root of *Codonopsis pilosula* (Franch.) Nannf. (*dǎng shēn*), 15g; the dried root of *Astragalus mongholicus* Bunge (*huáng qí*), 18g; the dried root of *Paeonia lactiflora* Pall. (*bái sháo*), 10g; the dried rhizome of *Coptis chinensis* Franch. (*huáng lián*), 6g; the dried sclerotium of *Poria cocos* (Schw.) Wolf (*fú líng*), 15g.	
Zhang 2015	/	The dried rhizome of Conioselinum anthriscoides 'Chuanxiong' (*chuān xiōng*), 5g; the dried rhizome of *Atractylodes macrocephala* Koidz. (*bái zhú*), 10g; the dried root and rhizome of *Salvia miltiorrhiza* Bunge (*dān shēn*), 15g; the dried root and rhizome of *Glycyrrhiza glabra* L. (*gān cǎo*), 10g; the dried bark of Neolitsea cassia (L.) Kosterm. (*ròu guì*), 20g.	Prepared by Zhang.
Guo et al. 2014	Radix Astragali and Galla Chinensis mixture (*Qí Bèi Hé Jì*)	The dried root of *Astragalus mongholicus* Bunge (*huáng qí*), 300g; the dried processed product of *Aconitum carmichaeli* Debeaux (*fù zǐ*), 60g; the dried root and rhizome of *Rheum palmatum L.* (*dà huáng*), 50g; the dried rhizome of *Zingiber officinale* Roscoe (*gān jiāng*), 30g; the insect gall on the leaf of *Rhus chinensis* Mill. (*wǔ bèi zǐ*), 50g; halloysite (*chì shí zhī*), 600g; the dried tuber of *Bletilla striata* (Thunb.) Rchb.f. (*bái jí*), 50g.	Prepared by Guo et al.
Xu 2013	*Xī Lèi Săn*	The dried processed product of leaf or stem and leaf of *Strobilanthes cusia* (Nees) Kuntze (*qīng dài*); the pearl produced by *Pteria martensii* (Dunker) (*zhēn zhū*); the	Prepared by Xu.

		crystals of the sulfate mineral mirabilite (*hán shuĭ shí*); the processed product of *Cinnamomum cam phora* (L.) Presl (*bīng piàn*); the dried gall-stone of Bos taurus domesticus Gmelin (*niú huáng*); the processed product by ripe fresh watermelon and mirabilite (*xī guā shuāng*). Making powder of the above medicinals, taking a total of 3g.	
Chen et al. 2010	*Chái Sháo Liù Jūn Kē Lì*	The dried root of *Bupleurum chinense* DC. (*chái hú*), 10g; the dried root of *Paeonia lactiflora* Pall. (*bái sháo*), 15g; the dried ripe peel of *Citrus* × *aurantium* L. (*chén pí*), 6g; the dried processed tuber of *Pinellia ternata* (Thunb.) Makino (*bàn xià*), 10g; the dried tuberous root of *Pseudostellaria heterophylla* (Miq.) Pax (*tài zĭ shēn*), 15g; the dried root and rhizome of *Glycyrrhiza glabra* L. (*gān căo*), 6g; the dried rhizome of *Atractylodes macrocephala* Koidz. (*bái zhú*), 15g; the dried sclerotium of *Poria cocos* (Schw.) Wolf (*fú líng*), 15g; the dried herb of *Scleromitrion diffusum* (Willd.) R.J.Wang (*bái huā shé shé căo*), 20g; the dried root and rhizome of *Panax notoginseng* (Burkill) F.H.Chen (*sān qī*), 10g; the dried herb of *Pteris multifida* Poir. (*fèng wĕi căo*), 20g.	Jiangyin Tianjiang Pharmaceutical Co., Ltd., China.
Zhang et al. 2010	Adjusted Ginseng, Poria and Atractylodes Macrocephalae Powder (*Shēn Líng Bái Zhú Săn*)	The dried root and rhizome of *Glycyrrhiza glabra* L. (*gān căo*); the dried rhizome of *Atractylodes macrocephala* Koidz. (*bái zhú*); the dried ripe seed of Dolichos lablab L. (*bái biăn dòu*); the dried sclerotium of *Poria cocos* (Schw.) Wolf (*fú líng*); the dried rhizome of *Dioscorea oppositifolia* L. (*shān yào*); the dried root and rhizome of *Panax ginseng* C.A.Mey. (*rén shēn*); the dried ripe seed of *Nelumbo nucifera* Gaertn (*lián zĭ*); The dried rhizome of *Dioscorea opposita* Thunb (*shān yào*); The dried ripe fruit of *Amomum villosum* Lour. (*shā rén*); the dried	Prepared by Zhang et al.

		ripe kernel of *Coix lacryma-jobi* L. (*yì yĭ rén*); the dried ripe fruit of *Ziziphus jujuba* Mill. (*dà zăo*); the dried rhizome of *Coptis chinensis* Franch. (*huáng lián*); the dried aboveground part of *Portulaca oleracea* L. (*mă chĭ xiàn*); the dried root of *Pulsatilla chinensis* (Bunge) Regel (*bái tóu wēng*); the dried aboveground part of *Pogostemon cablin* (Blanco) Benth (*huò xiāng*); the dried aboveground part of *Eupatorium fortunei* Turcz. (*pèi lán*); the dried bark of *Fraxinus chinensis* Roxb. (*qín pí*); the dried root of *Sanguisorba officinalis* L. (*dì yú*); the dried root of *Dolomiaea costus* (Falc.) Kasana & A.K.Pandey (*mù xiāng*); the dried fruit of *Prunus mume* (Siebold) Siebold & Zucc. (*wū méi*); the dried unripe fruit of *Citrus* × *aurantium* L. (*zhĭ qiào*).	
Gao et al. 2010	/	The dried root of *Codonopsis pilosula* (Franch.) Nannf. (*dăng shēn*), 15g; the dried rhizome of *Atractylodes macrocephala* Koidz. (*bái zhú*), 30g; the dried ripe seed of Dolichos lablab L. (*bái biăn dòu*), 15g; the dried ripe kernel of *Coix lacryma-jobi* L. (*yì yĭ rén*), 15g; the dried root and rhizome of *Salvia miltiorrhiza* Bunge (*dān shēn*), 10g; the dried rhizome of Coptis chinensis Franch. (huáng lián), 10g; the dried root and rhizome of *Glycyrrhiza glabra* L. (*gān căo*), 10g; the dried root of *Sophora flavescens* Aiton (*kŭ shēn*), 10g; the dried rhizome of Conioselinum anthriscoides 'Chuanxiong' (*chuān xiōng*), 10g; the processed product of of the dried rhizome of *Zingiber officinale* Roscoe (*páo jiāng*), 10g; the dried bark of Neolitsea cassia (L.) Kosterm. (*ròu guì*), 10g.	Prepared by Gao et al.
Lu et al. 2008	/	the resin of *Dracaena cochinchinensis* (Lour.) S. C. Chen (*lóng xuè jié*), 6g	Yunnan Datang Hanfang

	Pharmaceutical Co.,Ltd., China.
